# Modern slavery in the United Kingdom: The illegal migration act risks undermining efforts to combat exploitation

**DOI:** 10.1371/journal.pmed.1004279

**Published:** 2023-09-05

**Authors:** Sian Oram

**Affiliations:** Section of Women’s Mental Health, Institute of Psychiatry, Psychology & Neuroscience, King’s College London, London, United Kingdom

## Abstract

In this Perspective, Sian Oram discusses the potential implications of the Illegal Migration Act for victims of modern slavery in the United Kingdom.

## Teaser

The Illegal Migration Act, which recently passed through the United Kingdom (UK) parliament, poses a serious threat to the well-being of victims of modern slavery and efforts to combat exploitation. The Act gives the UK Government greater powers to deny support and allow the detention and deportation of potential victims and has been widely criticised, including by medical associations and charities. Measures included in the Act risk perpetuating the deprivation of safety, dignity, and medical care experienced by victims, instead of providing the protections to which they should be entitled.

Modern slavery is defined as the recruitment, movement, harbouring, or receiving of women, men, or children using force, coercion, abuse of vulnerability, deception, or other means for the purpose of exploitation. Victims endure prolonged and repeated acts of cruelty, abuse, neglect, and degradation and it remains a significant issue in the United Kingdom (UK). The Illegal Migration Act, which has recently passed through the UK Parliament, significantly diminishes key legal protections for victims of modern slavery, presenting a threat to their health and human rights [1].

Since the establishment in 2009 of the National Referral Mechanism (NRM), the framework by which potential victims of modern slavery are identified and supported in the UK, there has been a significant increase in the identification of modern slavery. In 2022, 16,938 potential victims were referred to the NRM—a 33% increase from the previous year [2]. Labour exploitation and criminal exploitation are the most reported forms of abuse among adults and children, respectively [2]. Despite Government concerns about people abusing the modern slavery system by posing as victims to prevent their removal from the UK, evidence does not suggest that this is a growing problem [3]. Indeed, it is believed that the number of victims going unidentified by authorities and support services remains high [4].

Healthcare settings are important sites for identifying and caring for victims of modern slavery. Research suggests a significant proportion of victims present to health services while being exploited [5], including emergency departments, maternity services, sexual health clinics, and general practice. Indicators in healthcare settings include signs of control, frequent address changes, neglect or poor living conditions, unexplained injuries and illnesses, sexual health concerns, and mental health problems [6]. Healthcare professionals can support disclosure by seeing patients alone, using independent interpreters where required, and following a trauma-informed approach. When modern slavery is suspected, offering choice, addressing immediate health needs, arranging follow-up care, and following local safeguarding procedures are essential. In cases of urgent safety risk, healthcare professionals should encourage the patient to remain in their care and contact the police [6].

Suspected victims of modern slavery can be referred into the NRM by designated First Responder agencies, including police, immigration enforcement, local authorities, and a small number of authorised charities, but excluding the NHS. Although details of how the Illegal Migration Act will be implemented are not yet clear, the Act allows for significant changes to NRM processes where a victim entered the UK illegally. Previously, referrals led to 2 consecutive decisions about victim status by Home Office decision-makers: the initial “reasonable grounds” decision and, if this first decision was positive, the later “conclusive grounds” decision [7]. A decision that there were reasonable grounds to suspect that a person may have been a victim of modern slavery granted access to government-funded support for a period of at least 30 days in England and Wales. Support should have included safe accommodation where required, financial support to meet essential needs, and practical help and advice from a support worker, including assistance to access healthcare and legal aid, and exemption from charges for NHS care. Where there were conclusive grounds that a person was a victim of modern slavery, victims were entitled to at least 45 days of “move-on” support in England and Wales and continued to be exempt from healthcare charges.

The Illegal Migration Act gives the UK Government greater powers to deny support and allow the detention and deportation of potential victims and follows a narrowing of victim protections by the earlier Nationality and Borders Act. Critics, including medical associations and charities, have raised concerns about the Illegal Migration Act’s compliance with human rights obligations and international law [1,8,9]. The Act significantly diminishes key legal protections for potential victims who have arrived irregularly in the UK (including those for whom entry into the UK was part of the criminal offence of trafficking committed against them), such as the right to remain in the country while awaiting a conclusive decision and to receive specialised support services [1] These measures risk perpetuating the deprivation of safety, dignity, and medical care experienced by victims, instead of providing the protections to which they should be entitled.

Immigration detention, and even the threat of detention and deportation, has detrimental effects on mental health [10,11], with victims of modern slavery at particular risk due to the already high prevalence of mental health issues and experiences of physical and sexual violence [12]. Healthcare services in immigration removal settings are largely inadequate and deporting individuals without proper safeguards increases the risk of future harm, including re-trafficking. Furthermore, denial of specialised services is likely to significantly hinder recovery due to the challenges of accessing healthcare and other essential services unassisted [5], such as providing necessary registration documents, navigating systems, accessing interpretation and translation services, and disclosing abuse. Accessing mental healthcare is especially challenging due to limited availability of trauma-informed therapies, long waiting lists [13], and the hesitancy of some mental health professionals to initiate therapies in the context of social and legal stressors [10].

The Illegal Migration Act poses a serious threat to survivor well-being and efforts to combat exploitation. A comprehensive and survivor-centred strategy for combating modern slavery requires the prompt determination of victim status, expanded long-term care and support, and improved training for professionals [14]. Survivors highlight secure housing, safety, long-term support, trauma-informed services, purpose in life, and access to medical treatment and education as being key to their recovery [15]. Strengthening the response to modern slavery requires prioritisation of the recovery and well-being of victims and restoration of the human rights and dignity of which they have already been deprived.
